# Adaptive Image Enhancement Using Entropy-Based Subhistogram Equalization

**DOI:** 10.1155/2018/3837275

**Published:** 2018-08-13

**Authors:** Liyun Zhuang, Yepeng Guan

**Affiliations:** ^1^School of Communication and Information Engineering, Shanghai University, Shanghai, China; ^2^Faculty of Electronic and Information Engineering, Huaiyin Institute of Technology, Huai'an, China; ^3^Key Laboratory of Advanced Displays and System Application, Ministry of Education, Beijing, China

## Abstract

A novel image enhancement approach called entropy-based adaptive subhistogram equalization (EASHE) is put forward in this paper. The proposed algorithm divides the histogram of input image into four segments based on the entropy value of the histogram, and the dynamic range of each subhistogram is adjusted. A novel algorithm to adjust the probability density function of the gray level is proposed, which can adaptively control the degree of image enhancement. Furthermore, the final contrast-enhanced image is obtained by equalizing each subhistogram independently. The proposed algorithm is compared with some state-of-the-art HE-based algorithms. The quantitative results for a public image database named CVG-UGR-Database are statistically analyzed. The quantitative and visual assessments show that the proposed algorithm outperforms most of the existing contrast-enhancement algorithms. The proposed method can make the contrast of image more effectively enhanced as well as the mean brightness and details well preserved.

## 1. Introduction

Image contrast enhancement technology is regarded as a classical and important area in image processing. It is widely used in daily photo enhancement, medical image analysis, remote-sensing imagery, microscopic imaging [1], and many other areas [2–6]. Histogram equalization (HE) [7] is most extensively utilized for contrast enhancement. Good contrast images should have the characteristic that the histogram uniformly distributes over the entire range of the intensity. The visual quality of the image is improved by the HE method based on that fact. HE stretches the dynamic range of the histogram by remapping the gray levels on the basis of probability density function (PDF) of the image. In general, the HE has the advantages of efficient computation, quick results, and the usage of real-time applications. Despite these advantages, the HE method has some undesirable effects such as saturation effect, overstretching of input intensities, and so on. It tends to lose the details of image, shift the mean of the input image irrespective of image contents, and disturb the brightness of the image [8].

Substantial HE-based approaches have been developed to overcome the shortcomings of the HE technique in the past decades. However, achieving an enhanced image with high quality in the field of image processing is still a challenging task. In order to more effectively increase the contrast of the input image with brightness and details well preserved, an efficient algorithm named entropy-based adaptive subhistogram equalization (EASHE) is developed in this paper. The proposed method is more effective for preserving the mean brightness and detailed information of the enhanced image while improving the contrast compared with some other state-of-the-art methods. According to the experimental results based on 100 images from CVG-UGR-Database for some state-of-the-art methods and our proposed method, we know that the EASHE technique can achieve the multiple objectives of entropy maximization, details, and brightness preservation as well as control on over enhancement. The main contributions of this paper are as follows: Firstly, we introduce the entropy value-based algorithm to divide the histogram of the input image. Secondly, a novel approach for dynamic range adjustment of image gray level is developed to overcome the grayscale merging and image detailed information missing problems. Thirdly, we put forward a new algorithm to adjust the probability density function of the gray level, which can adaptively control the degree of image enhancement, and the output image looks more natural and clearer. Furthermore, results indicate that the proposed method is a better approach compared with the state-of-the-art methods.

The remainder of this paper is organized as follows: In Section 2, we give an overview of the related work.  Section 3 presents the proposed EASHE method. Data samples and performance evaluations are drawn in Section 4.  Section 5 provides experimental results and comparisons with state-of-the-art methods, and our concluding remarks are included in Section 6.

## 2. Related Works

Several HE-based approaches have been reviewed in this section. In order to preserve the mean brightness of the image and improve the contrast, Kim [8] proposes an algorithm named brightness preserving bihistogram equalization (BBHE). It separates the input image histogram based on the input image mean value. DSIHE [9] utilizes input image median to segment histogram, and equal number of pixels are contained in each subhistogram. MMBEBHE [10] is the extension of the BBHE method that provides maximal brightness preservation, which recursively divides the image histogram into multiple groups based on mean brightness error (MBE). DHE [11] partitions histogram based on locations of minima present in the histogram. The span of gray levels in the enhanced image for each subhistogram is decided based on their span in the input image and cumulative frequencies. Though these methods can perform good contrast enhancement, they cause more annoying side effects, including failing with images having nonsymmetric distribution [8], failing to preserve mean brightness [9], producing more annoying side effects [10], and losing structural information [12]. In these techniques, however, the difference between input and output image is minimal, and the desired improvement may not always be achieved [13].

More recently, recursive mean-separate HE (RMSHE [14]) is proposed by Chen and Ramli. The RMSHE further divides the histogram into two parts recursively according to their respective mean value. Each subhistogram is equalized independently by performing BBHE [8], and output image is constructed by the union of all equalized subhistograms. The mean brightness of enhanced image approaches towards the mean brightness of the input image. Sim et al. present another recursively separated (RS) HE method known as recursive subimage HE (RSIHE [15]), which is similar to RMSHE proposed by Chen and Ramli in [14]. RSIHE divides the histogram of the input image based on median values, and 2^*r*^ subhistograms are generated, where each subhistogram has an equal number of pixels.

In addition to histogram segmentation (i.e., BBHE, DSIHE, RSIHE, etc.), in order to improve HE, histogram clipping also has been developed. Histogram clipping can reduce the domination effect of high frequency bins during HE by controlling the enhancement rate. Examples of histogram clipping-based methods developed by scholars include bihistogram equalization with a plateau limit (BHEPL) [16] and bihistogram equalization median plateau limit (BHEPL-D) [17]. BHEPL is the combination of BBHE and clipped HE. First, the input image is separated by using the mean brightness of image, and then the subhistograms are clipped by using their plateau limits. Then, these subhistograms are separately equalized. The BHEPL-D is similar to the BHEPL, and the difference is that the BHEPL-D clips each subhistogram based on the median of the occupied intensity in the subhistogram.

In [18], Singh et al. recently propose an image enhancement technique using exposure-based subimage histogram equalization (ESIHE). The ESIHE method clips the input histogram at the average number of intensity occurrences and segments the clipped histogram using a threshold based on the image exposure. Singh et al. present a recursive-division-based extension of ESIHE, referred as RS-ESIHE [19]. RS-ESIHE performs recursive divisions of the histogram based on the image exposure. The algorithms based on the recursive division may fail to give natural-looking results due to inappropriate subdivisions. Moreover, deciding the number of division is a critical issue, which may degrade the performance of the algorithm. Singh and Kapoor propose median mean-based subimage clipped histogram equalization MMSICHE [20] algorithm for image enhancement, which firstly performs histogram partition based on median intensity and then divides each subhistograms based on mean intensity.

Additionally, many researchers also propose other HE-based enhancement methods with contrast improvement and brightness and details preservation. For example, modified histogram equalization (MHE) is proposed by Abdullah-Al-Wadud [21]. The proposed MHE approach manipulates the accumulation in the input histogram components before equalizing the histogram. It focuses on preserving the small parts in images. The dynamic histogram specification introduced by Sun et al., which can preserve the shape of the input image histogram, unfortunately, makes limited contrast enhancement [22]. Tsai et al. developed a contrast enhancement algorithm for color images [23, 24]. Huang et al. proposed an adaptive gamma correction with weighting distribution (AGCWD [25]), which enhances the contrast and preserves the overall brightness of an image. In the algorithm, the probability distribution for luminance pixels and the gamma correction is used. The AGCWD approach may not give desired results while it may lose details in the bright regions of image when there are high peaks in the input histogram [26]. Bihistogram equalization using modified histogram bins (BHEMHB) was proposed by Tang and Isa [27], and the algorithm segments the input histogram into two subhistograms according to the median value of the image. BHEMHB alters the histogram bins before HE is applied, but unfortunately limited improvement of contrast is achieved.

## 3. Proposed Image Enhancement Method

### 3.1. Entropy-Based Threshold Calculation

The proposed approach provides an optimal division of the original histogram. It is achieved by performing division of the histogram based on the entropy. A subhistogram is divided into two subhistograms with equal entropy. The histogram of an image is divided into four parts with three thresholds which are adaptive and obtained by the same method. The procedure to obtain the thresholds will be presented in detail as follows:

Consider an input image *I* with intensity levels in the dynamic range of [*k*_l_, *k*_u_], and let *H*[*k*_l_, *k*_u_] be the global histogram of the input image *I*, where *k*_l_ and *k*_u_ denote lower and uppermost intensities of the image *I*. *H*(*k*) is the histogram of the gray level *k*, which is defined as

(1) $$\begin{array}{c} H\left(k\right)=n_{k}\text{,}\;\text{for}\;k=k_{l},\ldots ,\;\;k_{u}, \end{array}$$

where *n*_*k*_ is the gray level of *k* in the image *I*. The pdf of the image, pdf(*k*), can be described as

(2) $$\begin{array}{c} \text{pdf}\left(k\right)=\frac{H\left(k\right)}{N_{\text{um}}},\;\;\;\mathrm{for}\;k=k_{l},\ldots ,\;\;k_{u}, \end{array}$$

where *N*_um_ is the total number of pixels in the input image *I*.

The entropy of *H* can be represented as

(3) $$\begin{array}{c} E\left(H\right)=-\overset{k_{u}}{\underset{k=k_{l}}{\sum }}\text{pdf}\left(k\right)\;\log\;\;\mathrm{pdf}\left(k\right). \end{array}$$

The threshold value for histogram segmentation can be obtained: First, we divide the whole histogram into two parts by an adaptive threshold *k*_s_. Then, the two parts can be presented as Sub_0_{*k*_l_ ~ *k*_s_} and Sub_1_{*k*_s_+1 ~ *k*_u_}. The entropy of *H* can be calculated by

(4) $$\begin{array}{c} E\left(H\right)=-\overset{k_{u}}{\underset{k=k_{l}}{\sum }}\text{pdf}\left(k\right)\;\log\;\;\mathrm{pdf}\left(k\right). \end{array}$$

The intensity level *k*_s_ is obtained by solving

(5) $$\begin{array}{c} -\overset{k_{\text{s}}}{\underset{k=k_{l}}{\sum }}\text{pdf}\left(k\right)\;\log\;\;\mathrm{pdf}\left(k\right)=\frac{1}{2}E\left(H\right). \end{array}$$

We can obtain the threshold *k*_s_ by (5), which is utilized to segment the histogram of image. Note that we set *k*_s2_=*k*_s_, and the optimal thresholds *k*_s1_ and *k*_s3_ of the two parts up and down the threshold *k*_s2_ can also be obtained in the same way as the above. Finally, the histogram *H*[*k*_l_, *k*_u_] is segmented into four subhistograms, that is,

(6) $$\begin{array}{c} H\left[k_{l},k_{u}\right]=\overset{4}{\underset{i=1}{\cup }}H_{r}\left[k_{l}^{r,4},l_{u}^{r,4}\right], \end{array}$$

where *k*_l_^*i*,4^ and *k*_u_^*i*,4^ represent the boundary values of the luminance range within the *r*th segmentation. Hence, all subimages are captured by

(7) $$\begin{array}{c} I_{\text{sub}}^{r,4}=\left\{k\left(i,j\right)\left|k_{l}^{r,4}\leq k\right.\left(i,j\right)\leq k_{u}^{r,4},\;\forall \left(i,j\right)\in k\right\}. \end{array}$$

The input image can be represented as a combination of segmented subimages.

(8) $$\begin{array}{c} I=I_{\text{sub}}^{1,4}\;\cup \;I_{\text{sub}}^{2,4}\;\cup \;I_{\text{sub}}^{3,4}\;\cup \;I_{\text{sub}}^{4,4}. \end{array}$$

### 3.2. Segment-Dependent Range Allocation

**Figure 1:**
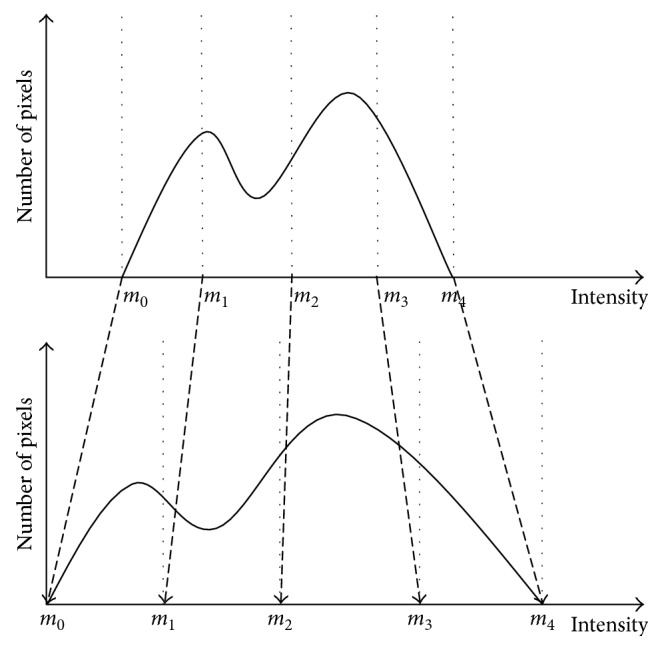
Grayscale remapping diagrammatic sketch.

In Section 3.1, the histogram of the original image is divided into four subhistograms based on the entropy. The gray level intervals are [*m*_0_, *m*_1_], [*m*_1_, *m*_2_], [*m*_2_, *m*_3_], and [*m*_3_, *m*_4_], respectively. Note that here *m*_0_=*k*_1_, *m*_1_=*k*_s1_, *m*_2_=*k*_s2_, *m*_3_=*k*_s3_, and *m*_4_=*k*_u_. Usually, most of the existing HE-based approaches equalize subhistograms independently within the original segmentation boundaries. Unfortunately, the HE over narrow ranged subhistograms (having separating points closer to each other) may result in saturation of intensities. On the contrary, HE over widely spaced subhistograms may give rise to uneven expansion of intensities. As a consequence, a resulted image may lose its natural appearance. Therefore, it is necessary to adjust the dynamic range of the subhistogram before the equalization. The process of adjustment is as follows:

(9) $$\begin{array}{c} {\text{span}}_{r}=m_{r-1}-m_{r},\\ {\text{aspan}}_{r}={\text{span}}_{r}+\left(E\left(H_{r}\right)-1\right)\;*\;\left(\left(\frac{L}{N_{\text{subs}}}\right)-{\text{span}}_{r}\right), \end{array}$$

where *L* is the number of gray levels (i.e., for 8 bits image, *L* = 256) and *N*_subs_ is the number of subhistograms. *E*(*H*_*r*_) is the entropy of the *r*th subhistogram, given as

(10) $$\begin{array}{c} E\left(H_{r}\right)=-\overset{k_{u}}{\underset{k=k_{l}}{\sum }}\text{pdf}\left(k\right)\;\log\;\;\mathrm{pdf}\left(k\right),\\ {\text{range}}_{r}=\frac{{\text{aspan}}_{r}}{\sum_{k=1}^{4}{\text{aspan}}_{r}}\left(L-1\right), \end{array}$$

where aspan_*r*_ denotes the grayscale range of the *r*th subhistogram in the input image histogram, *L* is the total gray level, and range_*r*_ represents the dynamic range of the *r*th subhistogram in the output image histogram. After adjusting the gray level dynamic range of subhistograms, the gray level range of the image is widely stretched, and the occurrence of grayscale combination is reduced, as shown in Figure 1. We can get the new boundary values of the luminance range within the *r*th segmentation formulated as

(11) $$\begin{array}{c} k_{l\_\text{new}}^{r,4}=k_{l\_\text{new}}^{r-1,4}+1,\\ k_{u\_\text{new}}^{r,4}=k_{\text{l}\_\text{new}}^{r,4}+{\text{range}}_{r}. \end{array}$$

### 3.3. Adaptive Probability Density Function Adjustment

The degree of image enhancement usually cannot be controlled by HE, so the phenomenon of over enhancement often exists. In this paper, we introduce a control factor of the image enhancement degree, which can adaptively control the degree of image enhancement. In order to simplify the calculation process, we divide the adjusted dynamic range histogram (processed in Section 3.2) into two subhistograms. The algorithm adjusting the probability density function of the gray level in this paper is given by (12).

(12) $$\begin{array}{c} {\text{pdf}}_{\text{EDADJ}}=\left\{\begin{array}{cc} {\text{pdf}}_{\text{avgmm}}-\alpha \frac{{\left({\text{pdf}}_{\text{avgmm}}-\text{pdf}\left(k\right)\right)}^{2}}{{\text{pdf}}_{\text{avgmm}}-{\text{pdf}}_{\min}}, & \;\text{pdf}\left(k\right)<{\text{pdf}}_{\text{avgmm}},\\ {\text{pdf}}_{\text{avgmm}}+\alpha \frac{{\left(\text{pdf}\left(k\right)-{\text{pdf}}_{\text{avgmm}}\right)}^{2}}{{\text{pdf}}_{\max}-{\text{pdf}}_{\text{avgmm}}}, & \;\text{pdf}\left(k\right)>{\text{pdf}}_{\text{avgmm}}, \end{array}\right. \end{array}$$

where pdf_max_ and pdf_min_ are the maximum and the minimum values of pdf, respectively, and pdf_avgmm_ is the average value of pdf_max_ and pdf_min_. The control factor *α* can be described as

(13) $$\begin{array}{c} \alpha =\left\{\begin{array}{cc} \frac{I_{\text{ks}}-I_{\text{avgsub}1}}{I_{\text{avgsub}2}-I_{\text{avgsub}1}}, & 0\leq k\leq I_{\text{ks}},\\ \frac{I_{\text{avgsub}2}-I_{\text{ks}}}{I_{\text{avgsub}2}-I_{\text{avgsub}1}}, & I_{\text{ks}}<k\leq L-1, \end{array}\right. \end{array}$$

where *I*_avgsub1_ and *I*_avgsub2_ are the average values of gray level in two subhistograms, respectively. They can be defined as

(14) $$\begin{array}{c} I_{\text{avgsub}1}=\frac{\sum_{k=0}^{I_{\text{ks}}}k\;*\text{ pdf}\left(k\right)}{\sum_{k=0}^{I_{\text{ks}}}k\;*\text{ pdf}\left(k\right)},\\ I_{\text{avgsub}2}=\frac{\sum_{k=I_{\text{ks}}+1}^{L-1}k\;*\text{ pdf}\left(k\right)}{\sum_{k=I_{\text{ks}}+1}^{L-1}k\;*\text{ pdf}\left(k\right)}. \end{array}$$

**Figure 2:**
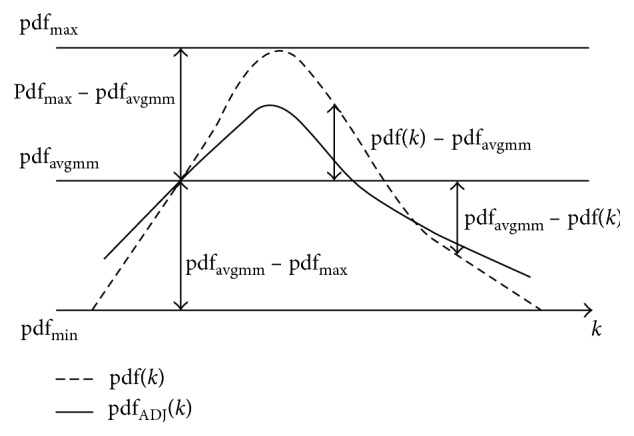
Diagrammatic sketch of probability density function adjustment.

*I*_ks_ is the intensity level obtained by solving

(15) $$\begin{array}{c} -\overset{I_{\text{ks}}}{\underset{k=0}{\sum }}{\text{pdf}}_{\text{ADJ}}\left(k\right)\;\log\;\;{\text{pdf}}_{\text{ADJ}}\left(k\right)=\frac{1}{2}E_{\text{ADJ}}\left(H\right), \end{array}$$

where pdf_ADJ_ is calculated after adjusting the gray level dynamic range of subhistograms in Section 3.2 and *E*_ADJ_ denotes the entropy value formulated by (16). The diagrammatic sketch of probability density function adjustment is indicated in Figure 2.

(16) $$\begin{array}{c} E_{\text{ADJ}}\left(H\right)=-\overset{L-1}{\underset{k=0}{\sum }}{\text{pdf}}_{\text{ADJ}}\left(k\right)\;\log\;\;{\text{pdf}}_{\text{ADJ}}\left(k\right). \end{array}$$

After adjusting the probability density function, it is necessary to normalize the cumulative distribution function. The process is as follows:

(17) $$\begin{array}{c} {\text{cdf}}_{\text{ADJ}}\left(k\right)=\overset{k}{\underset{i=0}{\sum }}{\text{pdf}}_{\text{ADJ}}\left(k\right),\\ {\tilde{\text{cdf}}}_{\text{ADJ}}\left(k\right)=\frac{{\text{cdf}}_{\text{ADJ}}\left(k\right)}{{\text{cdf}}_{\text{ADJ}}\left(k\right)\left(L-1\right)}\left(L-1\right). \end{array}$$

### 3.4. Histogram Equalization

HE involves mapping an input gray level *L* using transformation function *f*(*l*), which can be denoted as

(18) $$\begin{array}{c} f\left(k\right)=K_{l}+\left(K_{u}-K_{l}\right)\;*\text{ cdf}\left(k\right), \end{array}$$

where *K*_l_ and *K*_u_ represent the minimum and maximum gray levels, respectively. As observed in (18), the remapping of the input image is within the entire dynamic range [*K*_l_, *K*_u_] after applying CHE. The proposed method equalizes the modified subhistograms by (19), thereafter, the equalized subhistograms are integrated to produce the final enhanced output image.

(19) $$\begin{array}{c} f{\left(k\right)}_{{\text{sub}}^{r,4}}=k_{l\_\text{new}}^{r,4}+\left(k_{u\_\text{new}}^{r,4}-k_{l\_\text{new}}^{r,4}\right)\;*\;{\tilde{\text{cdf}}}_{\text{ADJ}}\left(k\right)\text{,}\;\text{for}\;\;k=k_{l\_\mathrm{new}}^{r,4},\ldots ,\;\;k_{u\_\mathrm{new}}^{r,4}. \end{array}$$

## 4. Data Samples and Performance Evaluations

### 4.1. Data Samples

The proposed approach is tested on 100 images from a public image database named CVG-UGR-Database [28]. The proposed approach is compared with conventional as well as state-of-the-art contrast-enhancement algorithms. The comparison algorithms are BBHE [8], DSIHE [9], RMSHE [14], MMBEBHE [10], RSIHE [15], DHE [11], BHEPL [16], ESIHE [19], MMSICHE [21], and BHEMHB [27]. The reason of choosing these methods for comparison is that they are mainly based on histogram segmentation, histogram clipping, and/or histogram modification. A comparative analysis of the different methods is performed by subjective and objective evaluation of the resulting images.

### 4.2. Subjective Evaluation

Subjective evaluation is a visual way to evaluate the contrast enhancement. The enhancement results can be appreciated if the enhanced image gives pleasurable effect in appearance. The judgment of annoying artifacts, over enhancement, and unnatural enhancement can be achieved by visual quality inspection. The performance of contrast enhancement algorithm can be effectively measured through the visual assessment results.

### 4.3. Objective Evaluation

The need for quantitative assessment arises due to the limitations of the human visual system. Moreover, human perception may sometimes be subjective, that is to say, enhancement or improvement of the visual quality of an image is a subjective matter because its judgment varies from person to person. Here, a qualitative analysis regarding the amount of details of the image, level of contrast, homogeneity of regions, and naturalness is performed, and we can establish numerical justifications by quantitative measurements. However, it is difficult to find an objective measure that is in accordance with the subjective assessment due to the lack of any universally accepted criterion. Here, we evaluate the performance of enhancement techniques using three quality metrics: discrete entropy (DE), peak signal to noise ratio (PSNR), and absolute mean brightness error (AMBE).

#### 4.3.1. Evaluation of the Richness of Information

Entropy is applied to measure the details in the image according to the Shannon theory [29]. Theoretically, the higher the entropy value, the greater the details contained in the image, so a higher entropy value is desired. The entropy value of the entire image can be calculated by the following:

(20) $$\begin{array}{c} \text{DE}=\overset{L-1}{\underset{l=0}{\sum }}e\left(l\right)=-\overset{L-1}{\underset{l=0}{\sum }}\text{pdf}\left(X_{k}\right)\;\log_{2}\text{ pdf}\left(X_{k}\right), \end{array}$$

where the pdf(*X*_*k*_) is the normalized probability of the *k*th gray level. The entropy of the image can achieve maximum value only when pdf(0)=pdf(1)=…=pdf(*L* − 1)=1/*L*.

#### 4.3.2. Evaluation of Contrast Enhancement

A good enhancement method should not only enhance the contrast of the image but also yield an image that owns a natural-looking in output image. The approach should not amplify the noise level during the enhancement process [30]. PSNR is commonly used to evaluate the quality achievement among the input and processed images [12, 31–34] and the degree of contrast enhancement in the image. MSE is firstly computed by (22), and then PSNR value is calculated in (21). Usually a large PSNR value is desired for the reason that the higher value of the PSNR indicates less significant noise level that is amplified. It means that the processed image is least degraded compared with the original input image. Mathematically, both of the quantitative measurements are given as follows:

(21) $$\begin{array}{c} \text{PSNR}=10\log_{10}\left[\frac{{\left(L-1\right)}^{2}}{\text{MSE}}\right], \end{array}$$

where MSE is the mean square error, defined as

(22) $$\begin{array}{c} \text{MSE}=\overset{i=I_{\text{width}}-1}{\underset{i=0}{\sum }}\overset{j=I_{\text{height}}-1}{\underset{j=0}{\sum }}\frac{{\left[X\left(i,j\right)-Y\left(i,j\right)\right]}^{2}}{I_{\text{width}}\times I_{\text{height}}}, \end{array}$$

where *I*_width_ and *I*_height_ represent the width and height of the images, respectively. *X*(*i*, *j*) and *Y*(*i*, *j*) are the input and enhanced image intensity value at the location (*i*, *j*) correspondingly.

#### 4.3.3. Evaluation of Brightness Preservation

In order to evaluate the ability of the proposed EASHE technique in mean brightness preservation, the objective function named average mean brightness error (AMBE) is utilized. AMBE is used to compute the difference of the mean brightness value between the input and enhanced images, as indicated in (23) [35–37]. Ideally, the mean brightness of the enhanced image should be equal to the mean brightness of the input image, therefore, a small AMBE is thus desired.

(23) $$\begin{array}{c} \text{AMBE}=\left|E\left(X\right)-E\left(Y\right)\right|, \end{array}$$

(24) $$\begin{array}{c} E\left(X\right)=\overset{i=I_{\text{width}}-1}{\underset{i=0}{\sum }}\overset{j=I_{\text{height}}-1}{\underset{j=0}{\sum }}X\left(i,j\right), \end{array}$$

(25) $$\begin{array}{c} E\left(Y\right)=\overset{i=I_{\text{width}}-1}{\underset{i=0}{\sum }}\overset{j=I_{\text{height}}-1}{\underset{j=0}{\sum }}Y\left(i,j\right), \end{array}$$

where *E*(*X*) and *E*(*Y*) are the mean brightness of the input and processed images, respectively.

## 5. Experiment Results and Discussion

### 5.1. Experiment Results

In this section, the simulation results of the proposed method EASHE are compared with state-of-the-art HE-based methods. As mentioned in Section 4.1, ten other HE-based techniques have been implemented to compare the performance of EASHE on contrast enhancement, brightness preservation, naturalness of the image, and ability to preserve details in the image.

**Figure 3:**
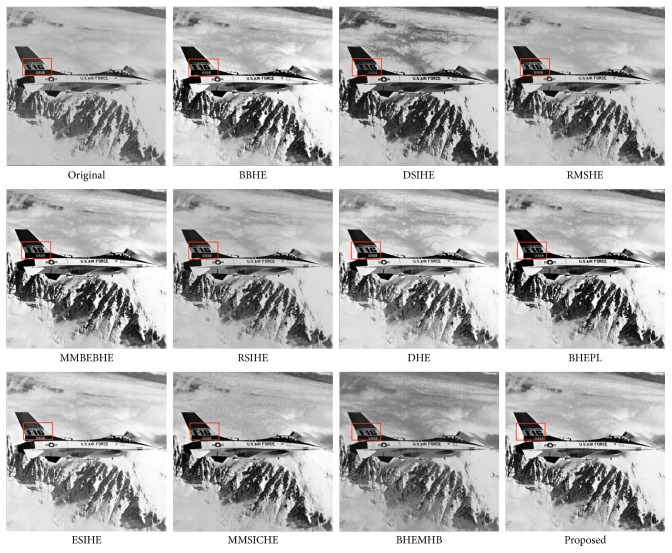
“F16” image and enhancement results of different algorithms.

**Figure 4 fig4:**
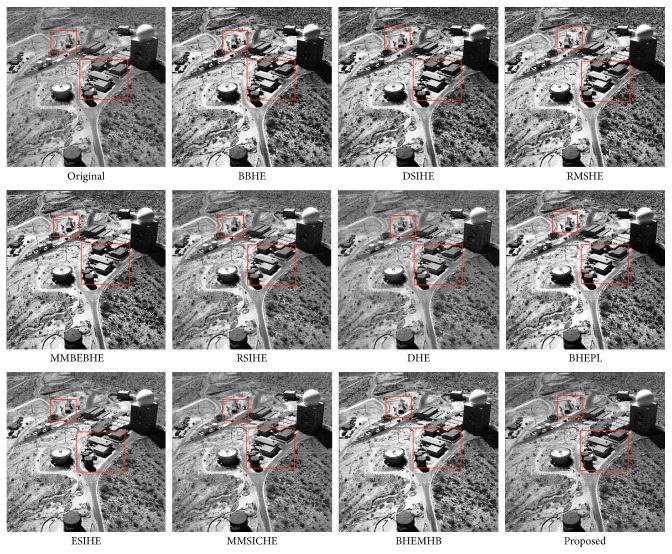
“Aerial” image and enhancement results of different algorithms.

**Figure 5 fig5:**
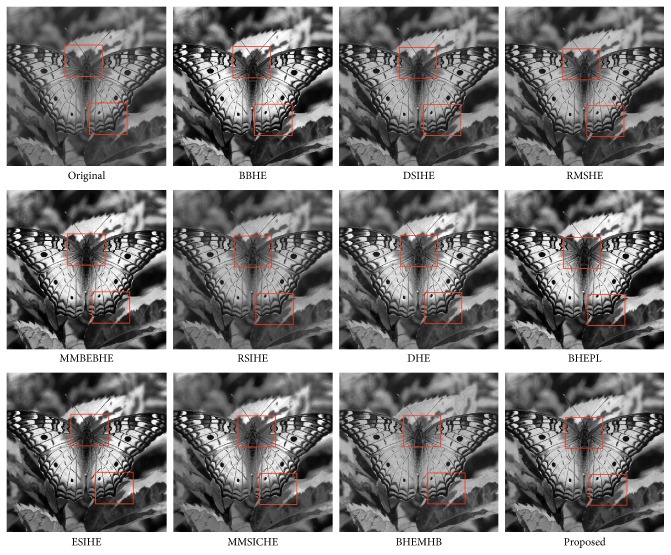
“Butterfly” image and enhancement results of different algorithms.

**Figure 6 fig6:**
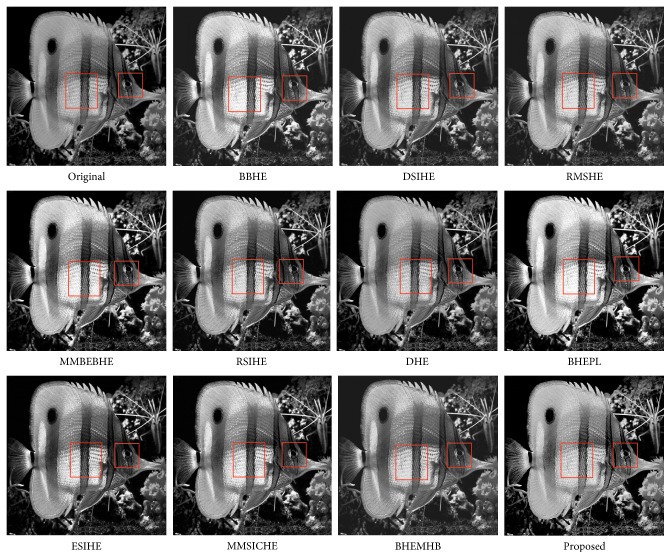
“Fish” image and enhancement results of different algorithms.

**Figure 7 fig7:**
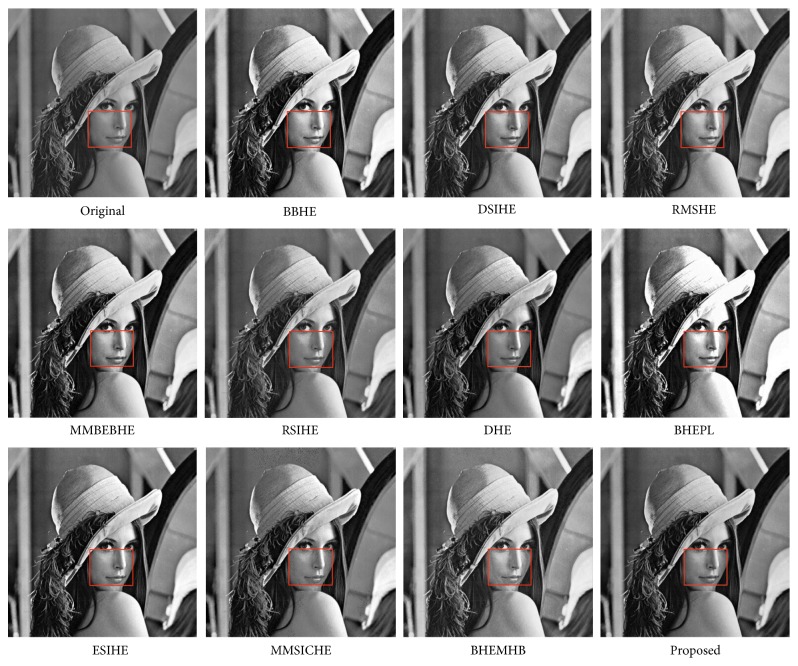
“Lena” image and enhancement results of different algorithms.

**Figure 8:**
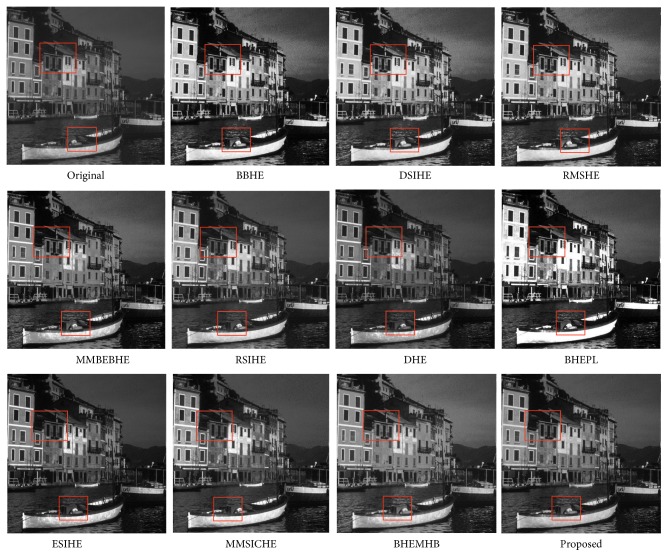
“Portofino” image and enhancement results of different algorithms.

In this article, the test images named as F16, Butterfly, Aerial, Fish, Lena, and Portofino are given. They are presented in this study for initial performance evaluation on our proposed EASHE method. The results obtained for each image are indicated in Figures 3 [Fig fig4] [Fig fig5] [Fig fig6] [Fig fig7]–8, respectively. “Original” indicates the input image, while the other images represent the respective enhanced images after applying other compared methods and our proposed EASHE approach. Tables 1 [Table tab2]–3 show the quantitative results of these test images. The best value for each analysis is in bold face.

Figure 3 shows the “F16” image and its contrast-enhanced results obtained by different algorithms. BBHE, DHE, and MMBEBHE enhance the contrast of the input image, but some regions exhibit over enhancement. Limited improvement of contrast enhancement is obtained by DSIHE and RMSHE. RSIHE, ESIHE, and BHEPL stand out some details, but the output image looks like dark due to the limited improvement in brightness. MMSICHE and BHEMHB can well preserve the brightness. The mean brightness of the enhanced image processed by proposed EASHE method is closest to the input image. So, the overall appearance of the processed image is very similar to the input image. The brightness can be well preserved in the processed image, since the proposed EASHE method can obtain the lowest AMBE value, as indicated in Table 3. The proposed method can grape the highest value of entropy, displayed in Table 1, which shows that most of the details of the image can be well preserved compared with the other methods. This can be seen from the highlighted area with red boxes. EASHE also produces images with homogeneous texture. Most of the image area, particularly the background of the image, appears to have a smooth texture with a few small regions. Our proposed technique least amplifies the noise level in the image during the enhancement process, since the largest PSNR value is obtained by the EASHE-ed image.

The “Aerial” image and its contrast-enhanced versions obtained by different algorithms are shown in Figure 4. BBHE and DHE methods make limited improvement on contrast-enhancement, and the BBHE obtains the lowest PNSR value, which is indicated in Table 2. There are some regions exhibiting over enhancement by BHEPL and RMSHE. MMBEBHE and MMSICHE can get a relative dynamic contrast than that of mentioned approaches, but some regions still look unnatural. The shifting effect of mean brightness is significant in the DSIHE-ed and ESIHE-ed images, which causes the loss of naturalness in these output images. Compared with the most of the other techniques, majority of the details of the image can be well preserved by EASHE, even though our proposed method is ranked second (i.e., 7.6931) after BHEMHB (i.e., 7.7350). The EASHE method least amplifies the noise level in the image during the enhancement process in that it can obtain the largest PSNR value (i.e., 26.1423), as shown in Table 2.

It can be observed on the window area highlighted with boxes that the proposed EASHE can simultaneously enhance the overall contrast of the test image “Butterfly” to an optimum level and preserve the details of the image, as shown in Figure 5. It is clear that the saturation effect is less apparent and thus the window areas can be clearly seen. This saturation effect (i.e., the window area regions become too bright) can be observed in the BBHE-ed and MMBEBHE-ed images. Observation on the ability of the proposed EASHE to preserve details is supported by the entropy measurement, in which the enhanced image has a entropy value larger than most of the methods, which indicates that the information entropy is well preserved. The EASHE-ed image has the largest value of PSNR (i.e., 29.3826), showing that the proposed method least degrades the image during the enhancement process. In addition, the EASHE-ed image has the lowest AMBE value (i.e., 1.7246), which indicates that the proposed method can well preserve the brightness of the output image.

The “Fish” image and its contrast-enhanced versions obtained by different algorithms are indicated in Figure 6. BBHE, MMBEBHE, and BHEPL introduce saturation effects in some regions in the output images, as shown in the highlighted areas with red boxes. DSIHE and RMSHE make limited improvement for contrast enhancement. RSIHE obtains a good contrast image. However, the image is slightly over enhanced. ESIHE and MMSICHE result in good contrast with natural visual quality. However, the proposed algorithm (EDSHE) provides better contrast enhancement. The EASHE-ed image has the largest value of PSNR (i.e., 28.6235), indicating that EASHE least degrades the image during the enhancement process. The EASHE method can simultaneously enhance the overall contrast of the “Fish” image to an optimum level and well preserve the detailed information. This outcome can be observed on the fish scale highlighted with boxes. As shown in Table 1, the proposed EASHE grapes the biggest value of entropy (i.e., 7.1325), indicating that the detailed information is well preserved. Furthermore, the proposed EASHE method can get the lowest AMBE value, which demonstrates the brightness can be well preserved in the processed image. The output image enhanced by EASHE, as shown in Figure 6 (proposed), also exhibits a natural look, which means it does not look too artistic after the enhancement process.

Figure 7 displays the contrast-enhancement results for the “Lena” image. The input image Lena has the characteristics that fully black or fully white regions are relatively few, as shown in Figure 7 (original). We can observe that some region of face is over enhanced by BBHE, MMBEBHE, and BHEPL methods. DSIHE, RMSHE, and RSIHE fail to significantly improve contrast. The resultant image enhanced with the proposed EASHE has a clearer contour compared with images using the other methods, as can be seen on regions within boxes. The proposed EASHE ranked first place for test image “Lena” in the entropy measurement, which indicates that more detailed information can be preserved in processed image by our method. The difference reveals that the performance of EASHE is comparable with others in retaining detailed information of enhanced image. Furthermore, the proposed method can well preserve brightness because it can get the lowest AMBE value. The proposed algorithm can obtain a natural-looking contrast-enhanced image.

The “Portofino” image and its contrast-enhanced images obtained by different algorithms are displayed in Figure 8. For the test image, the proposed EASHE produces an output image with most of the details well preserved because it possesses the highest entropy value. This result can be seen on regions highlighted with boxes, such as edges of the building and some regions of the boat. The shifting effect of mean brightness is pregnant in the MMBEBHE-ed and MMSICHE-ed images, resulting in the loss of naturalness in these images. RMSHE, RSIHE, and DHE fail to achieve much improvement of contrast enhancement. Some regions of the processed image exhibit over enhancement with BHEPL approach. By contrast, the resultant image enhanced with EASHE has a smooth texture, wherein less nonhomogenous regions are observed compared with other techniques. In addition, the EASHE-ed image has the smallest AMBE measurement.

**Figure 9:**
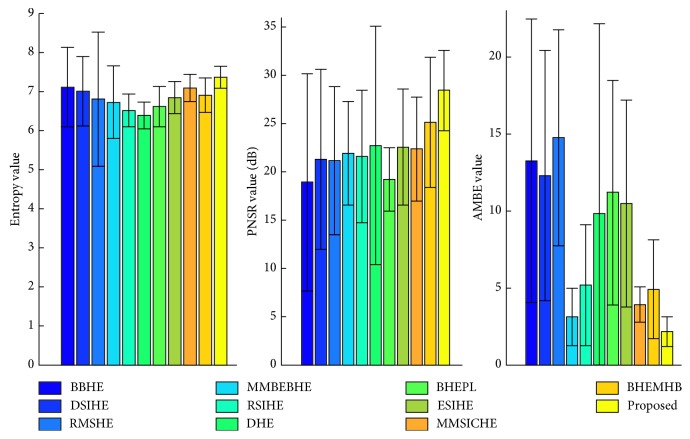
Average values and standard deviations of quantitative analyses for 100 test images.

From the performance of the proposed technique for the six test images, namely, F16, Butterfly, Aerial, Fish, Lena, and Portofino, it is clear that our proposed algorithm can obtain satisfactory results when compared with those of the other ten HE-based methods. In order to further justify the capability and performance of our EASHE method, we further validate the performance of the proposed approach with the three objective evaluation functions (i.e., entropy, PSNR, and AMBE) by utilizing the 100 test images from CVG-UGR-Database. The average values and standard deviations of these quantitative analyses for 100 test images are presented in Figure 9.

As indicated in Figure 9, our proposed ESAHE technique illustrates outperformance when compared with the state-of-the-art HE-based methods. On average, the image processed by EASHE contains the highest amount of details. The richness and detail information can be well preserved in output image due to its highest entropy value (i.e., 7.37). The proposed EASHE method outperforms all the other algorithms compared in this paper. The largest PSNR (i.e., 28.42) value gained by our method indicates that the output images processed by EASHE have a more natural appearance with minimum artifacts compared with others. The lowest value (i.e., 2.16) obtained by our method shows that the enhanced image using EASHE has mean brightness nearest to the original image.

### 5.2. Discussions

The highest PSNR value by the EASHE-ed image indicates that the proposed EASHE method can enhance the image with minimum noise and artifacts. It illustrates that the contrast enhancement performance of EASHE is better than most of state-of-the-art HE-based methods. With regard to mean brightness, the EASHE-ed image demonstrates high capability, especially when compared with the BHEPL-ed images. The enhanced image by BHEPL algorithm is too bright when referred to the original image. The naturalness of the image is maintained in the EASHE-ed image, because the degree of image enhancement is adaptively controlled. The image is enhanced at a sufficient level without introducing an unpleasant look while improving the contrast of the input image.

Furthermore, AMBE values for all the techniques are computed. EASHE acquires the lowest AMBE value compared with the other methods. The EASHE possesses the highest capability in retaining the mean brightness of the image due to its lowest AMBE value, that is to say, the images processed by EASHE typically have a mean brightness closest to the original image.

## 6. Conclusion

A new approach named the entropy-based adaptive subhistogram equalization (EASHE) with brightness and detailed information preservation is presented in this paper. The presented approach recursively separates the input histogram based on the entropy value of histogram. The proposed method provides a better distribution of intensity levels over the entire dynamic range, which results in an effectively incensement of contrast. The detailed information can be well preserved by utilizing a novel algorithm to adjust the probability density function of the gray level. The proposed algorithm is compared with some state-of-the-art HE-based algorithms, and a large number of images from standard image database are used to test the performance of the proposed approach. The experimental results have shown that the EASHE method can obtain superior performance compared with some HE-based state-of-the-art methods.

## Figures and Tables

**Table 1:** DE calculated for the test images.

Image	BBHE	DSIHE	RMSHE	MMBEBHE	RSIHE	DHE	BHEPL	ESIHE	MMSICHE	BHEMHB	Proposed
F16	6.3327	6.3590	6.0909	6.5023	6.4910	6.5124	6.5438	6.6120	6.6026	6.6660	**7.4261**
Aerial	7.0015	6.9640	7.9891	6.9567	7.0971	7.1369	6.9752	7.5100	7.6289	**7.7350**	7.6931
Butterfly	7.1428	7.2368	7.1569	7.0623	6.9257	7.2463	6.9674	7.0680	7.0816	6.8156	**7.1520**
Fish	6.0326	6.1604	6.1689	6.2672	5.9100	6.2672	6.1324	5.9850	6.1247	6.0170	**7.1325**
Lena	7.2324	7.1996	7.0085	7.2195	7.4610	7.5984	7.5124	7.4970	7.5128	7.5620	**7.8361**
Portofino	6.5736	6.4447	6.2251	6.3516	6.5054	6.4368	6.3018	6.4836	6.3927	6.6470	**6.7100**

**Table 2 tab2:** PSNR calculated for the test images.

Image	BBHE	DSIHE	RMSHE	MMBEBHE	RSIHE	DHE	BHEPL	ESIHE	MMSICHE	BHEMHB	Proposed
F16	18.3412	20.9870	21.8760	24.4849	22.1130	20.7826	21.6876	22.8690	23.6437	23.9420	**27.5627**
Aerial	18.3726	19.7078	19.3277	24.0514	24.4368	24.1023	23.8672	23.6780	25.2106	24.4635	**26.1423**
Butterfly	23.7126	23.2012	24.9133	23.5086	27.2658	26.7627	26.3412	27.9320	28.0131	28.2672	**29.3826**
Fish	21.3122	20.1459	19.7673	22.0862	24.9780	27.1671	23.2674	25.4060	26.0126	26.4010	**28.6235**
Lena	23.1417	23.5663	22.1785	22.7730	24.8000	27.9726	23.2672	25.7990	27.1211	26.5950	**30.2516**
Portofino	19.1426	21.7635	22.6540	25.3268	21.3264	25.1324	20.2673	22.2420	24.3746	24.5865	**26.3410**

**Table 3:** AMBE calculated for the test images.

Image	BBHE	DSIHE	RMSHE	MMBEBHE	RSIHE	DHE	BHEPL	ESIHE	MMSICHE	BHEMHB	Proposed
F16	1.3826	20.2554	5.7030	0.4496	6.4810	2.3726	0.5126	2.8740	2.7427	1.3340	**0.1257**
Aerial	2.1127	3.0264	1.9858	**0.7146**	1.0718	1.0172	1.3826	2.4570	1.3826	2.1652	2.0326
Butterfly	2.0134	1.8726	2.6479	2.1850	3.6592	3.1436	3.4131	3.6864	2.0131	3.1150	**1.7246**
Fish	7.2436	9.5402	11.7333	8.2674	3.7260	4.3672	3.1236	3.8960	2.3826	4.5760	**2.1026**
Lena	3.1427	6.0463	10.3377	0.8662	5.1090	2.1146	3.3324	2.5310	1.1324	2.4650	**0.7726**
Portofino	6.9624	14.6670	11.2179	2.3521	9.1660	7.3826	7.6423	10.2240	5.7624	8.1610	**1.3624**

## Data Availability

The data used to support the findings of this study are available from the corresponding author upon request.
